# Novel Personalized Dietary Treatment for Autism Based on the Gut-Immune-Endocrine-Brain Axis

**DOI:** 10.3389/fendo.2019.00508

**Published:** 2019-08-13

**Authors:** Ceymi Doenyas

**Affiliations:** Research Center for Translational Medicine, Koç University, Istanbul, Turkey

**Keywords:** autism, autism spectrum disorder, diet, personalized, immune, endocrine

## Abstract

Autism spectrum disorder (ASD) is a neurodevelopmental condition manifesting with impaired social interaction and communication, and restricted and repetitive behaviors and interests. In this perspective article, a more comprehensive approach than the gut-brain axis, hereby termed the “gut-immune-endocrine-brain” axis, is taken, based on which a personalized treatment plan for ASD is presented. ASD has no known etiology or cure, making desperate parents willing to try any treatment that worked for an individual with ASD, without much regard for its effectiveness, safety or side effects. This has been the case for restrictive dietary interventions as gluten-free/casein-free and ketogenic diets and recently, probiotics have emerged as the new such fad. One of the concerns about these dietary and probiotic treatments is their non-specificity: they may not be effective for all individuals with ASD, not all probiotic strains may have the beneficial qualities advertised indiscriminately for probiotics, and strains conferring benefits in one condition may not be probiotic in another. Not all children with ASD show immune reactivity to dietary proteins in wheat and milk, and wheat and milk may not be the only dietary elements to which reactivity is exhibited, where dietary aquaporins that resemble human aquaporins may elicit antibody reactivity in genetically susceptible individuals, which may include individuals with ASD. These observations are utilized to formulate a three-step plan to create effective, targeted, personalized treatments with as few side effects as possible, enabled by a systems approach connecting the various findings for dietary, immune, and neuroautoimmune reactivity in individuals with ASD.

Autism spectrum disorder (ASD) is a neurodevelopmental difference where affected individuals manifest impaired social interaction and communication, and restricted and repetitive behaviors and interests (1). Large cohort twin studies show that both genetic and shared and non-shared environmental factors contribute to ASD (2, 3), though a clear etiology is not known.

For devastating neurological conditions like ASD, it has been suggested that any simple lifestyle choice that could prevent or arrest neuroautoimmune reactivity should be investigated, such as the controllable choice of diet (4). There have been years of discussions about the administration of gluten-free/casein-free (GF/CF) diets and ketogenic diet (KD) and more recently of probiotics for ASD. The problem with these dietary recommendations is that they are made at a general level for all individuals with ASD.

This non-personalized approach that consider all individuals on the spectrum to have the same constellation of metabolic, endocrine, and physiological alterations is one of the reasons underlying the inconclusive evidence for their effectiveness, where some studies show improvement in ASD symptoms after GF/CF diet administration (5–9), and others do not (10–12). Similarly, though KD was shown to improve sociability and communication and decrease self-directed repetitive behavior in a genetic mouse model of ASD (13), in a human trial, some participants were not able to tolerate the KD and of the remaining group, only 60% showed improvement in ASD symptoms, which ranged from minor to significant improvement (14). These findings suggest that these diets may not be effective for all individuals with ASD and some may even experience discomforting outcomes that make them discontinue the regimen, which is the issue that this perspective article aims to address.

This article presents a novel, personalized dietary treatment regimen that will (1) avoid the difficulty and the potentially harmful effects of limiting certain nutrients unnecessarily and (2) create a targeted treatment for the individual immunological and endocrine profile of each patient of this disorder for which there is yet no cure.

This perspective does not undermine the possibility that GF/CF diet and KD may benefit some individuals with ASD, as was shown in a study that found both diets to result in significant improvement in ASD symptoms in a group of 15 participants, and KD to yield better cognition and sociability results compared to GF/CF diet (15). Instead, it argues that these generalized dietary plans of GF/CF diet and KD do not benefit all individuals with ASD given the different physiological profiles of each individual, and that as certain negative effects of these diets have started to emerge with recent investigations (16), they should not be recommended to all individuals with ASD without an initial screening and should be replaced by individualized dietary plans that fit their specific profile the best.

Such an idea to make alterations to these traditional diets have recently been embraced by other researchers as well. One trial tested a modified ketogenic gluten-free diet with the addition of MCT supplement and found certain improvements on core autism symptoms except for restricted and repetitive behaviors (17). Another trial tested a gluten-free, casein-free, soy-free diet with the addition of special vitamin/mineral supplements including essential fatty acids and digestive enzymes and observed improvements in ASD symptoms of 67 children and adults with ASD, yet some participants also experienced sickness, intestinal symptoms, mild nausea, loose stools, facial rash, worsening behaviors, increased aggression, and increased spinning behavior (18). These findings attest to the necessity of the perspective presented here that a simple modification of GF/CF and ketogenic diets with the addition of dietary supplements may not be sufficient and a profiling of each patient and the creation of personal diets may be what is needed to avoid these negative experiences and ensure the maximal benefit and satisfaction from dietary treatments.

Upon observing that the GF/CF diet resulted in greater improvements in children with ASD who had gastrointestinal symptoms, food allergies, and food sensitivities, Pennesi and Klein (19) suggested that gastrointestinal and immune factors may differentiate diet responders from diet non-responders. We build on this perspective by taking a personal approach to these immunological parameters, and aim to provide a system to discover the particular dietary regimen to which each individual with ASD will respond the most.

## Gut-Immune-Endocrine-Brain Axis Model for Autism

Throughout the years, external agents have been implicated in ASD and investigated mainly via comparisons of their levels in the body systems of or utilization by individuals with ASD and general population, such as pesticides (20, 21), toxic metals (22–24), and antibiotics (25, 26). These past propositions have remained at a speculative level owing to an absence of a delineated mechanism for their effect at the time. They recently gained traction with the discovery that these toxicants selectively target ASD genes (27) and the understanding of the different metabolic, signaling, immune-inflammatory functions of the gut microbiota on muscles, liver, and the brain (28), resulting in an increasing research focus on the involvement of the gut-brain axis in various neurological disorders including ASD (29).

With such a “paradigm shift in neuroscience” (29), previous findings including but not limited to the following started being investigated under the umbrella of the gut-brain axis: differences in urine peptides (30), intestinal permeability (31), gastrointestinal problems in individuals with ASD [which are not only experienced more often but also correlate with ASD severity (32)], and improvements in ASD behavior due to dietary changes. By suggesting a connection between the gut and the brain in ASD (33), this axis offers an explanatory model to link such diverse findings from individuals with ASD.

Though much speculation and theoretical perspectives have been offered about the involvement of the gut-brain axis in ASD in the past decade, recently emerging causal evidence is what brings a more solid grounding to this approach. Firstly, transplantation of gut microbiota from human donors with ASD induced core ASD behaviors in mice, whose brains showed alternative splicing of ASD-related genes (34). This finding supports not only a causal role for microbiota in the emergence of ASD symptoms but also a connection between the microbiota in the gut and ASD risk genes in the brain. Secondly, this evidenced connection between the gut and the brain was further clarified by another recent study. It found that probiotic rescue of social deficits in ASD mouse models happened via the vagus nerve and oxytocinergic and dopaminergic signaling in the brain (35). Thirdly, probiotic treatment rescued social deficits in genetic, environmental, and idiopathic mouse models of ASD (35, 36), and restored synaptic, in addition to social, deficits induced by maternal high-fat diet in mice offspring (37). These findings implicate probiotics as a promising treatment for many ASD cases with different etiologies. These are exciting developments, as the initial findings from 6 years ago of probiotic rescue of ASD symptoms (38) did not reveal the mechanism of action for this improvement, and recent evidence for probiotic effectiveness in different etiological ASD models and specific pathways for their effects bring scientific credence to the potential therapeutic value of gut-brain investigations in ASD.

More recently, another dimension was added to this axis given the aberrant immune responses and inflammatory profiles widely reported in ASD (39, 40), yielding the “gut-immune-brain” axis (41–43). The only three papers referencing this axis in relation to ASD consider inflammation and immune reaction in a systematic fashion and in general terms, where one presents a systematic review of gut-immune-brain mechanisms in ASD (41), the other investigates the role of sex in this communication (42), the latter suggests amino acids as a potential treatment to reduce inflammation and alter gut microbiota composition (43). I believe that a more specific approach to immunity that considers to which compounds that immune reaction is given in each individual with ASD is warranted, and this idea forms the basis of the treatment proposed in this paper.

Given the ability of the microbiota to produce psychoactive chemicals that are hormones or have hormonal qualities and considering the gut microbiota as a “unique collective endocrine organ,” Obrenovich et al. (44) have recently put forth the term “microbiota-gut-brain-endocrine interactome” based on the concept of an interactome denoting the complete molecular interactions in a biochemical system. Though they have created this term to explain the co-metabolism in human hosts in general, it is applicable to ASD well. Here, I wish to combine this terminology with the immune domain and conceive a “gut-immune-endocrine-brain interactome” on which I base my personalized treatment approach.

The bidirectional signaling between the gut and the brain is believed to happen through four main signaling pathways, which are the neural pathway including the vagus nerve enabling the gut microbiota to influence the brain and central nervous system efferent neurons that influence the gut; endocrine pathway involving enteroendocrine signaling stimulated by bacterial byproducts and hypothalamic-pituitary-adrenal (HPA) axis activation modulating the gut microbiota; neurotransmitters including serotonin of which about 95% is produced in the gut and those released by sympathetic activation and/or anti-inflammatory reflexes that influence the gut; and immune signaling from the reactions of the gut defensive barrier to externally derived pathogens and internal agents (45–47). While within the gut-brain axis, immune and endocrine functions are considered simply as pathways enabling communication between the gut and the brain, the new naming used here of the gut-immune-endocrine-brain axis additionally considers the influences of immune and endocrine mechanisms on the brain and neurological pathology.

## Probiotics

Probiotics, live microorganisms that provide a health benefit to the host when given in adequate amounts (48), are enjoying popularity as a potential treatment avenue for ASD, supported by initial hopeful findings of normalizations in gut microbiota alterations, improvements in gastrointestinal symptoms, and decreases in autism symptoms and severity upon probiotic administration in individuals with ASD (49–51).

In a previous paper, I have noted that the holistic physiological effects of dietary interventions are mostly unknown and though GF/CF diet and KD may benefit some subgroups of individuals with ASD, they have certain negative gut microbial, gastrointestinal, and metabolic effects. So, I suggested probiotics as a safer alternative than these restrictive diets since they assist gluten digestion, counteract gluten's harmful effects on gut permeability, reduce gut inflammation, increase gut integrity, and improve gastrointestinal and ASD symptoms (16). I concluded by suggesting the possibility that specific strains or combinations of probiotics may work better for individuals with ASD with specific co-morbid conditions, such as dietary protein intolerance. Nonetheless, while discussing the potential mechanisms of effect via which probiotics can serve similar benefits as restrictive diets without their harmful effects, I have not specified the probiotic strains that possess the specific features I talk about. Therefore, I may have fallen into the same fallacy that I have criticized above of considering probiotics in general terms, which I endeavor to rectify herein.

In reaction to misrepresentations about probiotics in the media, industry, and scientific field and generalized statements overlooking their limitations, a recent appeal was made to strictly observe the scientific definition and avoid generalizations from studies of single probiotic products (52). These authors reiterate the requirements to call an organism probiotic, such as being alive in adequate numbers, identified genetically and classified with latest terminology, and proven effective with appropriately sized and designed studies (52). They also note the importance of defining the basis for population stratification when designing individualized therapies (52), which is done in the present paper with clear screening procedures.

Similarly, a different group set out to show how such glorifications of probiotics as panaceas are indeed too good to be true. They found that probiotics create an individualized effect on gut bacteria and mucosal community structure, and based on these findings, they call for the development of new personalized probiotic approaches (53). This parallels the views of another group who note the dramatic increase in scientific, public, and industrial interest in probiotics and prebiotics as potential management agents for gut microbiota, and suggest that this field has the potential to create a new route for personalized medicine (54).

Following these appeals for personalized approaches and for avoiding generalized, non-specific references to probiotics, I present a novel, personalized dietary treatment based on the gut-immune-endocrine-brain axis for autism spectrum disorder.

## Novel, Personalized Dietary Treatment for ASD

### Background

Dietary proteins and interventions have been proposed to affect ASD symptoms through the gut-brain axis.

Panksepp (55) observed that behavior induced by low doses of narcotics resembled ASD behaviors, and proposed an excessive or unusual activity in endogenous brain opioid systems of children with ASD. Subsequently, an opioid-excess theory of autism was proposed. According to this theory, in individuals with ASD, peptides with opioid activity are formed from dietary sources, especially from those containing gluten and casein. These peptides pass through an abnormally permeable gut membrane and enter the central nervous system to influence neurotransmission and other physiological symptoms (56).

The underlying microbiota-gut-brain axis mechanisms of GF/CF diets have also been related to the premises of the opioid-excess theory (57). The increased endogenous opioids in the body fluids of individuals with ASD have been connected to altered activity in ASD of the DPP4 enzyme, which is able to decrease endogenous and exogenous opioid peptide levels in the blood (58). These excessive amounts of opioid peptides are suggested to pass the blood-brain barrier, bind to opioid receptors, and result in ASD symptoms. As gluten and casein are the precursors for these opioid peptides, removing them is proposed to improve ASD symptoms by positively influencing neurotransmission in the brain either directly or indirectly (58).

However, it is not clear whether this mechanism is at work in all individuals with ASD. This idea is supported by a randomized, double-blind, placebo-controlled trial administering a digestive enzyme supplement, which did not find any significant clinical improvement of ASD symptoms (59). This suggests that not all individuals with ASD may have abnormal digestion of gluten and casein leading to opioid peptides. Similarly, children who responded to a GF/CF diet were observed to give a heightened immune response to cow milk protein and/or gliadin (60). So, it is possible that not all individuals with ASD may have digestive problems with or immune reactivity to cow milk protein and gliadin. Therefore, individual measurements should be taken before recommending a GF/CF diet, which may not provide any behavioral benefits for some individuals with ASD and even be another negative factor aggravating social isolation (12).

Besides the elevated inflammatory reaction to dietary proteins observed in some individuals with ASD, it was also found that a subgroup of children with ASD produce antibodies against gliadin peptides and Purkinje cells (61). This finding is important for two reasons. First, it supports the existence a subgroup, and not all measured individuals with ASD, to have an immune reaction to gluten and gliadin, and it does so via another measurement besides pro-inflammatory cytokines, that of antibodies. Such antibody production may be another reason why GF/CF diets resulted in behavioral improvements for some ASD patients but not all of them. This finding attests once more to the need for individualized dietary treatment regimens for ASD. Secondly, the finding of a significant percentage of children with ASD showing elevated antibodies against not only gliadin but also cerebellar peptides suggests that the aberrant immunoreactivity in some individuals with ASD may not be limited to dietary proteins. Though the authors of this study suggest that these responses may be responsible for some neurological symptoms in ASD, the mechanism behind this and how such autoimmunoreactivity relates to aberrant immune reactions to dietary elements was not revealed until very recently.

Though it was not specifically tested in individuals with ASD, the very recent investigation into the similarities between dietary and human aquaporins may connect findings about dietary protein reactivity and autoimmunity in ASD. Aquaporins are membrane channel proteins that are found in plants and humans, including the gut barrier and the blood-brain barrier. Lambert et al. (4) showed that because of the shared structural homology between plant and human aquaporins, antibody immune reactions against food aquaporins can lead to neuroautoimmune reactivity. An already impaired intestinal barrier such as that suggested for ASD or a compromised gastrointestinal barrier due to reactivity with aquaporins in the gut barrier would allow antigenic material from foods and gut bacteria into the blood. Excessive antibody formation to these products may then trigger systemic inflammation and autoimmune reactivity, which may in turn affect the blood-brain barrier integrity, allowing circulating antibodies to enter the brain and target neurological tissues that resemble the antibody's target food antigen (4). As authors suggest that this environmentally induced neuroautoimmunity begins with genetic susceptibility for neurological disorders, this may be one model to connect genetic susceptibility and environmental triggers in ASD and the pathway underlying the previously mentioned finding of elevated antibodies against gluten and cerebellar cells in a group of children with ASD.

### From Evidence to Treatment

Two suggestions come from abovementioned researchers that can help guide the creation of personalized treatments for ASD. Jyonouchi et al. (60), upon discovering that the subgroup of ASD children who clinically responded to GF/CF diet had an increased production of proinflammatory cytokine TNF-α with cow milk protein and/or gliadin, suggest that these responses to dietary proteins may be a simple, objective marker to assess the presence of dietary protein intolerance in children with ASD. Lambert et al. (4) propose that practitioners working with patients with neurological conditions should test for food aquaporin reactivity, and if they obtain a positive result, should remove the offending foods, which may ameliorate the patient's clinical condition.

These dietary recommendations may nicely combine with prebiotic and probiotic components. One group who found increased mycotoxin levels in individuals with ASD and especially of the mycotoxin Ochratoxin A (OTA) suggested a personalized diet coupled with probiotics for OTA-positive individuals with ASD (62). Another group combined an exclusion diet with prebiotics. Prebiotics are non-digestible food ingredients that are fermented by probiotics, resulting in health benefits to the host by selectively stimulating these beneficial bacteria and in the production of short-chain fatty acids (SCFAs) that can diffuse into the blood, enabling a distal effect on numerous body organs (63). As the administration of prebiotics and an exclusion diet resulted in improvements in social behavior, gut microbiota, and metabolites in children with ASD, the authors propose that multiple interventions, such as their combination, may be more relevant to improve both physiological and psychological traits in ASD (64).

Taking these novel findings and recommendations by researchers into account, I present a personalized dietary treatment regimen based on the gut-immune-endocrine-brain axis and the specific immune reactivity and gut microbial profile of each individual on the autism spectrum.

**Figure 1:**
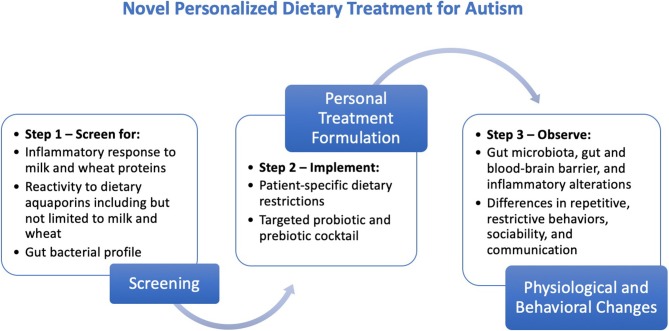
Steps for the creation, implementation, and evaluation of the proposed personalized dietary treatment regimen for individuals with ASD.

This proposed personalized treatment comprises the following three steps (Figure 1).

Step 1: Screening

- Screen for inflammatory response to milk and wheat proteins [see procedure by Jyonouchi et al. (60)]
- Screen for reactivity to dietary aquaporins including but not limited to milk and wheat [see procedure by Lambert et al. (4)]
- Perform fecal or another mapping of gut bacterial profile.

This preliminary proposition focused on milk and gluten proteins, since the currently available evidence suggests them to be the peptides against which children with ASD show higher levels of immunoglobulin antibodies (61, 65). In children with ASD, the antibodies against milk and gliadin peptides of gluten react with cerebellar peptides, which is not the case for antibodies for corn and soy, pointing to the significant antigenic cross-reactivity of gluten and milk with cerebellar antigens (61, 65). If future studies reveal similar antigenic cross-reactivity effects or increased immunoglobulin levels in individuals with ASD for other dietary proteins, they can also be included in the screening process.

Step 2: Implementation

Create a patient-specific dietary restriction plan based on inflammatory and aquaporin reactivity results from the screening.

Complement with prebiotic supplements [the most common prebiotics are Fructo-oligosaccharides (FOS), galacto-oligosaccharides (GOS), and trans-galacto-oligosaccharides (TOS) (63)] and targeted probiotic strains that address the microbial imbalance in the gut [see procedures by Tomova et al. (49) and Shaaban et al. (50)].

Though two studies normalized altered gut microbiota in individuals with ASD using probiotic supplements, a complete mapping of the effects of these used and other probiotics should be done before administration or recommendation. Through the ingestion of appropriate probiotic strains or prebiotic growth substrates for the beneficial bacteria (63), gut microbial imbalance in individuals with ASD can be restored.

Here, the focus should be on correcting altered levels of gut microbial strains that produce critical SCFAs, such as butyrate, which is recently implicated in the mechanism of non-allergic gluten/wheat sensitivity via its effects on a chain reaction of events in the gut (66). The results of future investigations along these lines in individuals with ASD may necessitate the addition of screening for the levels of important SCFA-producing gut bacteria to Step 1 in the forthcoming refinements of the present proposal.

When creating the probiotic and prebiotic cocktail, attention should be paid to the recently published reiteration that in order to be considered probiotic, microbial strain designation and at least one human study are needed (52). Additionally, if a strain is shown to be effective for a certain function, such as improving the gut barrier, then that strain is expected to confer the same benefit when administered in the presence of other strains, at its previously tested dose (52). This notion supports the utilization of more than one probiotic strain in this cocktail depending on the specific needs of the individual.

Step 3: Evaluation

Test for the gut microbial, gut and blood-brain barrier-related, and inflammatory changes in individuals with ASD via measurements before and after treatment.

Observe differences in repetitive/restrictive behaviors, communication, and sociability in individuals with ASD using measurements as Childhood Autism Rating Scale (CARS), Autism Diagnostic Observation Schedule (ADOS), and Autism Treatment Evaluation Checklist (ATEC).

It should be noted that this is a preliminary description of this novel personalized dietary treatment approach to ASD. Its aim lies more in the dissemination of this perspective than in the creation of a solid protocol, which will hopefully be the next step. Following investigations will evaluate the effectiveness and feasibility of such personalized screenings and the resulting dietary regimens for individuals with ASD of all ages, both sexes, and different levels of condition severity.

## Conclusion

This perspective article translates the current emphasis on the gut-brain axis and appeal for personalized interventions into a feasible step-by-step application to create personalized dietary treatments for individuals with autism. By following a more integrated gut-immune-endocrine-brain axis model, it explains why and how to formulate an individualized dietary plan that matches the specific inflammatory and aquaporin reactivity responses of each individual with ASD. This way, individuals with ASD can avoid the harmful gut microbial, gastrointestinal, and metabolic effects of GF/CF and ketogenic diets delineated previously (16) and receive targeted treatments for their specific physiological profiles. More investigations into the full spectrum of effect of particular probiotics and prebiotics will help flesh out a protocol to include specific recommendations for individuals based on their personal needs. This next step of nutritional neuropsychopharmacology (67) is what will enable the formulation of patient-specific treatment regimens with utmost effectiveness and minimal side effects for individuals with ASD.

## Author Contributions

CD conceptualized and wrote the manuscript.

### Conflict of Interest Statement

The author declares that the research was conducted in the absence of any commercial or financial relationships that could be construed as a potential conflict of interest.
